# Obstructive Sleep Apnea and Role of the Diaphragm

**DOI:** 10.7759/cureus.29004

**Published:** 2022-09-10

**Authors:** Bruno Bordoni, Allan R Escher, Anastasia Toccafondi, Luca Mapelli, Paolo Banfi

**Affiliations:** 1 Physical Medicine and Rehabilitation, Foundation Don Carlo Gnocchi IRCCS/Institute of Hospitalization and Care With Scientific Address, Milan, ITA; 2 Anesthesiology/Pain Medicine, H. Lee Moffitt Cancer Center and Research Institute, Tampa, USA; 3 Cardiology, Foundation Don Carlo Gnocchi IRCCS/Institute of Hospitalization and Care With Scientific Address, Milan, ITA; 4 Cardiopneumology: Cardiology, Foundation Don Carlo Gnocchi IRCCS/Institute of Hospitalization and Care With Scientific Address, Milan, ITA; 5 Cardiopneumology, Foundation Don Carlo Gnocchi IRCCS/Institute of Hospitalization and Care With Scientific Address, Milan, ITA

**Keywords:** osa, myofascial, copd, tongue, fascia, diaphragm, obstructive sleep apnea

## Abstract

Obstructive sleep apnea (OSA) causes multiple local and systemic pathophysiological consequences, which lead to an increase in morbidity and mortality in patients suffering from this disorder. OSA presents with various nocturnal events of apnoeas or hypopneas and with sub-clinical airflow limitations during wakefulness. OSA involves a large percentage of the population, particularly men, but the estimate of OSA patients could be much broader than data from the literature. Most of the research carried out in the muscle field is to understand the causes of the presence of chronic nocturnal desaturation and focus on the genioglossus muscle and other muscles related to dilating the upper airways. Sparse research has been published regarding the diaphragm muscle, which is the main muscle structure to insufflate air into the airways. The article reviews the functional anatomy of the muscles used to open the upper respiratory tract and the non-physiological adaptation that follows in the presence of OSA, as well as the functional anatomy and pathological adaptive aspects of the diaphragm muscle. The intent of the text is to highlight the disparity of clinical interest between the dilator muscles and the diaphragm, trying to stimulate a broader approach to patient evaluation.

## Introduction and background

Obstructive sleep apnea (OSA) is a respiratory disorder that negatively interferes with the phases of sleep, completely, partially, or intermittently interrupting the eupneic act. OSA can have central or peripheral origins. Injuries to the central nervous system can cause a functional limitation of the respiratory drive, with a total or partial decrease in diaphragmatic movement; peripheral obstructions may lead to the collapse of the pharyngeal airway, reducing the amount of inhaled air into the lungs [1]. A study that examined.1,520 asymptomatic adults in the United States found a percentage of peripheral OSA in about 15% in men and about 5% in women [2]. A recent study performed in Europe, with 1288 participants, found a peripheral OSA finding rate of about 30% in men and about 13% in women [3]. In the pediatric population, the finding of OSA is approximately 2-4% [4]. There are multiple risk factors for developing peripheral OSA. The presence of asthma and asthma medications can increase or develop collapsibility, or narrowing of the upper airways [5]. Asthma can lead to obesity and gastroesophageal reflux, the latter two being factors risk for the evolution of OSA [6]. A retrospective study has shown that the coexistence of asthma and OSA increases the rate of hospitalization for the acute exacerbation of asthma, compared to the co-presence of other diseases [7]. Increased bronchial hyperresponsiveness and the presence of systemic inflammation could be the most relevant causes of exacerbation in the presence of OSA and asthma [8]. Gastroesophageal reflux disease (GERD) can cause laryngeal dysfunction, leading to peripheral OSA, although the precise causes are elusive [9,10]. GERD does not exacerbate OSA and is not correlated with the severity of respiratory indices [11]. Obesity predisposes the onset of OSA (and obesity-hypoventilation syndrome), altering the muscular anatomy of the upper respiratory tract with greater nocturnal collapse; the systemic inflammatory environment related to obesity is linked as one of the leading causes of asthma and GERD, creating a vicious circle [6,12,13]. Most patients diagnosed with OSA are overweight (BMI 25.0-29.9) or obese (BMI>30) (approximately '80%) [14]. The systemic inflammation found in allergic rhinitis and chronic sinusitis triggers the same upper respiratory tract dysfunction mechanisms present in asthma, predisposing the onset of OSA [13]. Other causes can induce the presence of peripheral OSA. An observational study has highlighted that bilateral mandibular fracture can lead to the development of OSA while surgery to improve the orthopedic picture of the bone is able to induce the disappearance of OSA, probably due to purely mechanical factors [15]. We know that morphological and functional craniofacial alterations can predispose to the onset of peripheral OSA. Hypomobility or retrusion of the mandible, a structural alteration of the maxillary bone (hypoplasia), a non-physiological (lower) position of the hyoid bone, and an extended postural attitude of the cervico-occipital tract create the conditions for the finding of OSA. Through cephalometric measurements, it has been shown that patients with OSA have a higher vertical measurement in the facial area (tendentially), compared to people without OSA [16]. OSA can induce the onset of various co-morbidities with an increase in the mortality rate. Sleep apnea causes sleep fragmentation and chronic intermittent hypoxia; the frequency and severity of apneas/hypopneas are measured with the apnea-hypopnea index (AHI) while the level of desaturation is measured with the oxygen desaturation index (ODI) [17,18]. The presence of OSA with a high AHI and a severe ODI induces a decline in testosterone levels (particularly in middle-aged men), an altered pituitary-gonadal axis, with evidence of sexual dysfunction [19]. Hypogonadism may be associated with the presence of obesity in patients with OSA [20]. Regardless of hypogonadism, OSA can induce metabolic syndrome, diabetes, and obesity. OSA can activate a non-physiological metabolic environment, leading to a metabolic syndrome with diabetes mellitus, insulin resistance with pancreatic beta cell dysfunction, hypertension and dyslipidemia, cardiovascular disease, and obesity. Approximately 40% of patients with obesity develop peripheral OSA while approximately 70% of patients with OSA are obese. Metabolic syndrome can develop regardless of body mass index (BMI) and obesity [21]. There are several factors proposed to explain the onset and connection with OSA and the resultant metabolic and cardiovascular diseases (heart attack, atherosclerosis, atrial fibrillation, heart failure) in patients. OSA can induce an increase in the activity of the sympathetic system, create a systemic inflammatory environment (elevation of inflammatory cytokines and leptin), a higher generation of cellular oxidation; increases the activity of the renin-angiotensin system [22-24]. OSA increases the risk of long-term all-cause mortality, compared to the absence of OSA, by approximately 46%-54% more and approximately 1.9 times in patients with severe OSA [25]. OSA increases the finding of pulmonary hypertension (from 17% to 53%), probably, due to hemodynamic changes related to sleep apnea (pulmonary vasoconstriction and endothelial dysfunction) [22]. OSA can exacerbate acute episodes in patients with co-presence of chronic obstructive disease (COPD); the finding of OSA and COPD is referred to as overlapping syndrome and affects 1.0%-3.6% of the general population [26]. Acute exacerbation of OSA-COPD can involve about 61.4% of affected patients, with an increased mortality rate on first admission [26]. The cardiovascular and metabolic problems deriving from the presence of OSA are also reflected in the brain. There is a reduction in the thickness of the gray matter at the cortical level (paracentral and precentral anterior lobule) [27]. There is a cognitive decline, neuropsychological alterations, anxiety, and depression; such events are probably related to hypoxia with a decrease in glucose synthesis, an increase in deposits of beta-amyloid peptides (β-amyloid or Aβ), and a decline in cleansing by the glymphatic system [21]. The repeated desaturation of oxyhemoglobin during sleep apnea leads to a greater onset of stroke and cerebrovascular damage (optic and otological neuropathy) for about 61.9% of patients [22]. The anatomical areas on which the clinician's attention focuses in the presence of OSA are the upper airways, in particular, the lingual complex [28-30]. An anatomical area that very often does not attract the clinician's attention is the diaphragm muscle, despite the eupneic act involving multiple anatomical areas (from the nose to the pelvic floor) through a common central pattern generator (CPG) [31,32]. The article reviews the functional anatomy and adaptation in patients with OSA of the respiration muscles of the upper airways, and the functional anatomy and adaptations caused by sleep apnea in the diaphragm muscle, trying to emphasize the importance of the latter, in order to obtain a clinical picture that is as complete as possible.

## Review

Functional anatomy and adaptation of the upper respiratory tract with OSA

**Figure 1:**
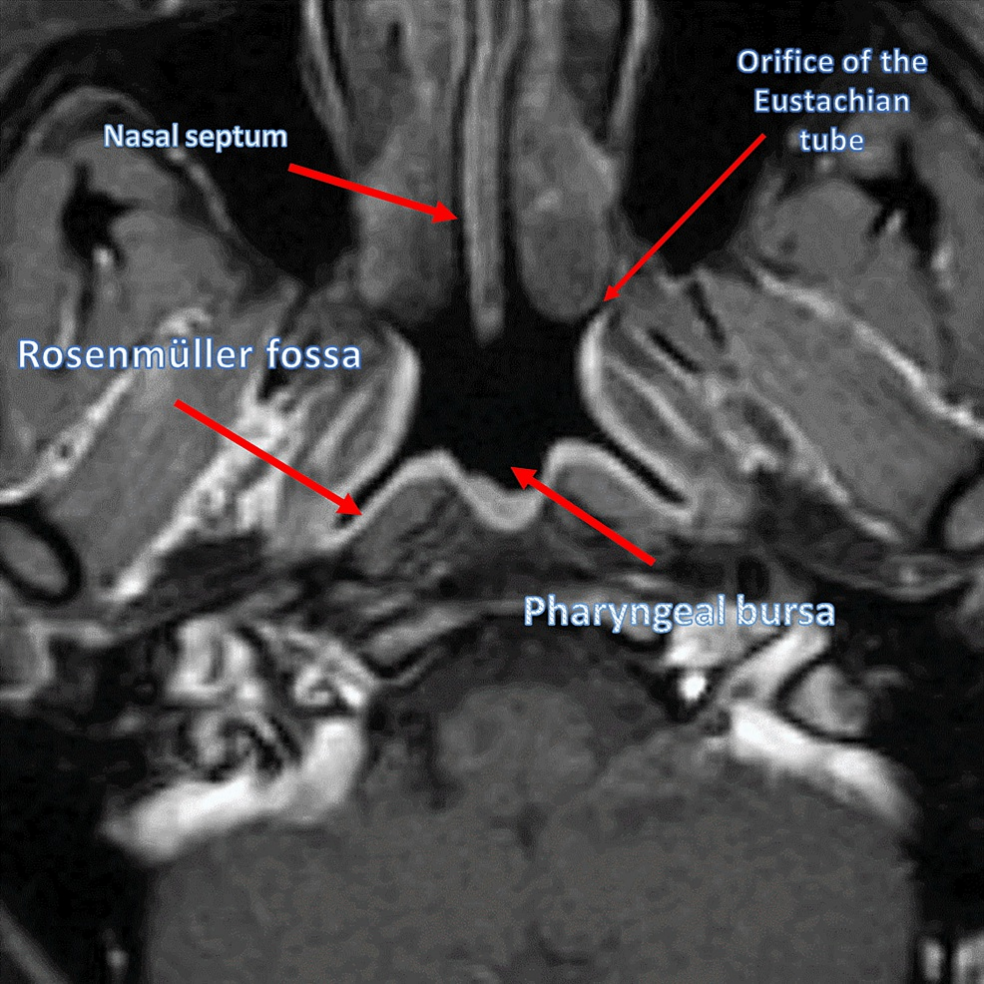
Magnetic resonance imaging of a healthy subject, highlighting certain portions of the nasopharynx, such as the orifice of the Eustachian tube, the pharyngeal bursa, and the Rosenmüller fossa, and the nasal septum (slightly deviated) The photo is owned by Bordoni Bruno.

All respiratory, secondary, and accessory muscles are managed by a central pattern generator (CPG), consisting of a neural network between the brain stem and the spinal cord where, in particular, we find important areas, such as the pre-Bötzinger complex, nucleus tractus solitarius (or nucleus of the solitary tract), ambiguous nucleus, parabrachial/Kölliker-Fuse complex, raphe-pontomedullary network, medullary division of the lateral tegmental field, and nucleus subcoeruleus [31]. Several other neurological areas intervene to coordinate the final act of breathing such as the cranial and spinal nerves, cerebellum, cortical and subcortical centers, and midbrain [31]. CPG controls 700 breaths per hour in a state of rest in a healthy person. We can divide the eupneic act into three distinct phases: preinspiratory, inspiratory, and expiratory. To the latter, two other phases are added, such as the post-inspiratory phase (extending the inspiratory phase) and the active expiratory phase [31]. The alae nasi or dilator naris is an extradiaphragmatic inspiratory muscle innervated by the facial nerve, which affects nasal airflow by opening the nasal passages and decreasing airflow resistance (stiffening the airways and preventing collapse) [32,33]. The airflow passes from the nasopharynx (or rhino-pharynx) to the oropharynx and then to the hypopharynx; these latter anatomical areas may become obstructed in patients with peripheral OSA [34]. The posterior area of the nasopharynx consists of loose connective tissue, in which area we can find some important structures such as the Eustachian tube, the pharyngeal bursa, and the Rosenmüller fossa (or lateral pharyngeal recess) [35]. Alae nasi is activated with the same timing activation of the genioglossus muscle and always before the diaphragm muscle in healthy subjects [36,37]. With OSA patients, electromyographic values increase, reducing latency; muscle contraction increases with increasing apnea episodes with a minor contractile pause between contractions, until awakening [38]. The dilator muscles of the airways in OSA patients, such as the alae nasi, are more active during wakefulness than in healthy subjects [39]. Dysfunction of this muscle can alter negative air pressure flows to the pharyngeal compartment, stimulating airway collapse [39]. We do not know how alae nasi fits metabolically and phenotypically in the presence of constant apneas. The nasopharynx area can undergo stenosis due to the increase of fibrotic tissue due to iatrogenic causes, in particular, due to previous surgery (and radiotherapy) at this site, causing the appearance of OSA over time (Figure 1) [40]. The resolution is surgical. In patients with OSA, it is possible to find a restriction of this area for various pathogenic causes, without reaching complete stenosis [41].

**Figure 2:**
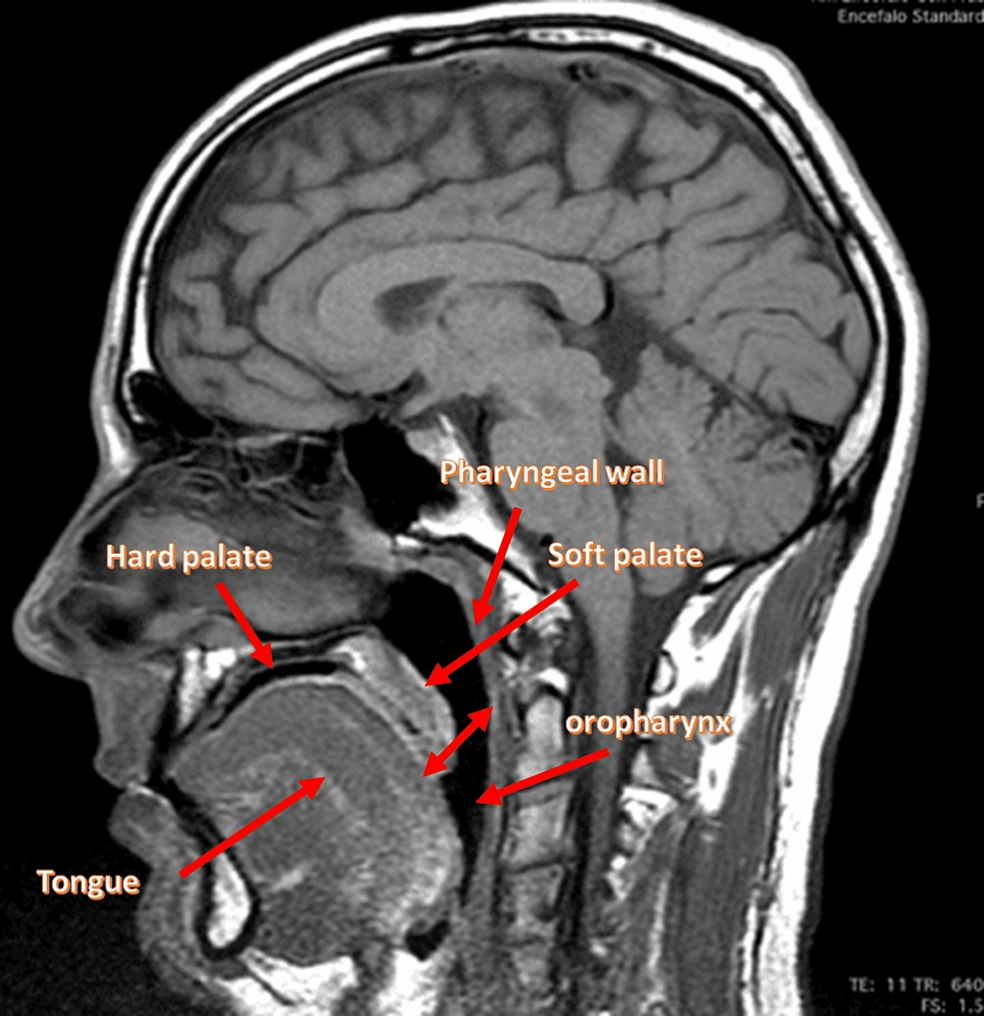
The magnetic resonance image highlights some anatomical components of a healthy person: the oropharynx; pharyngeal wall; tongue; soft palate (the double-headed arrow recalls the movement of the soft palate); and the hard palate The photo is owned by Bordoni Bruno.

The nasopharynx is located between the pharyngeal vault (non-muscular non-collapsing upper end of the nasopharynx) and the upper face of the soft palate, putting the nasal cavities in continuity with the oropharynx [35]. The soft palate is made up of five muscles covered by mucus membranes: tensor veli palatini (TVP), levator veli palatini (LVP); musculus uvulae or uvular muscle (UM); palatoglossus (PG), and palatopharyngeus (PP) [42,43]. The flow of air entering the nasopharynx is affected by TVP; the latter merges its aponeurosis with the uvula, the PP, and the PG and has rhythmic activation with the phases of the breath. In the animal model, TVP is able to influence the pharyngeal lumen at the level of the soft palate, but in the human model, we do not have a definitive result [42]. The innervation of the TVP derives from the maxillary branch of the trigeminal nerve [44]. LVP comprises about 40% of the length of the soft palate and is located between the hard palate and the uvula [42]. This muscle is longer in men than in women, but the action remains the same: raise and pull the soft palate, closing the wall pharyngeal posteriorly [42]. The activation of the LVP facilitates the work of the genioglossus muscle during respiratory acts, in synchrony with the alae nasi and palatoglossus [36]. Motor branches for LVP arise from the lesser palatine nerve (trigeminal nerve), and from the pharyngeal plexus [44]. We do not know the specific adaptation of this muscle in the presence of OSA. The contraction of UM allows to shorten the uvula, creating a convex shape to the velum, giving rigidity and extending the same velum toward the posteriority; UM is innervated by the lesser palatine nerve [44,45]. UM crosses and interconnects dorsally with the LVP and may consist of a single muscle medially or of bundles of muscles placed in parallel [45]. UM is capable of increasing its contractile capacity if the LVP undergoes a functional decrease, thus preserving the velopharyngeal closure [45]. In patients with OSA, UM undergoes non-physiological adaptations, altering its correct functionality. Cytoskeletal proteins, such as desmin (intermediate filament) and dystrophin (sub-sarcolemmal protein) undergo a reduction in their presence, negatively contributing to the contractile capacity of the muscle, compared to healthy subjects [46]. Muscle fibers change morphology, with a not as homogeneous form, with an increase in the connective tissue between the fibers; neurotrophin levels increase, such as brain-derived neurotrophic factor (BDNF), whose values indicate neuromotor remodeling processes in the presence of constant lesion [47]. UM undergoes a decrease in the capillary network and in the number of mitochondria, with decreased oxidative capacity; these adaptations lead to faster fatigue [48]. Muscle fibers exhibit an inflammatory environment in individuals with OSA, with increased levels of tumor necrosis factor (TNF)-alpha [49]. PG constitutes the soft palate below and is an integral part of the extrinsic muscles of the tongue, influencing its complex function and shape; it is founded on some intrinsic muscles of the tongue (superior longitudinalis and transversalis muscles) [50]. The contraction of the PG muscle allows bringing the soft palate toward the lingual complex (downward), in order to pass the food bolus correctly, reducing the measure of the oropharyngeal isthmus [50]. It constitutes the palatoglossal arches in the lateral pharyngeal area, preventing saliva from entering the oropharyngeal region (coming from the vestibular area) [50]. PG intervenes in the pronunciation of some phonemes through the deformation of the veil and the tongue [51]. It works with PP to create a sling effect during phronesis so that the oropharyngeal isthmus behaves like a sphincter [52]. PG would act as an antagonist to LVP [50]. Innervation is uncertain; depending on the literature, it could derive from the glossopharyngeal nerve, vagus nerve, or hypoglossal nerve [39,42,44]. During inspiration, it is activated in myoelectric coordination with the alae nasi, LVP, tongue, and diaphragm [36]. In patients with OSA, PG has a reduced myofibrillar diameter size and reduced capillary and mitochondrial density; fibrotic and heavy chain myosin fibers with non-specific type isoform are found [48]. These adaptations mirror a muscle that undergoes continuous remodeling in the presence of neuromuscular lesions, such as in dystrophies [48]. PG appears to undergo a more non-physiological adaptation. This is important as compared to UM [48]. Generally, in healthy subjects, PG and TVP have a balance in the distribution of oxidative and glycolytic fibers while there is a predominance of white fibers for MU and PP; LVP is rich in red fibers [53]. If this phenotypic physiological distribution in the muscles of the soft palate is altered, the function assigned to each district and the functions expressed in a group context will inevitably change. No muscle ever acts alone [44]. PP is a broad and flat muscle below the PG muscle, which connects the pharyngeal area with the soft palate; it coordinates with the LVP and the superior constrictor muscle of the pharynx (SCP) [42]. Together with the SCP, PP forms Passavant's ridge during the phase of velopharyngeal closure (during swallowing and phronesis) [54]. There is fusion in the lower portion of the salpingopharyngeal and PP muscle, and with the stylopharyngeal muscle at the level of the thyroid cartilage [55]. It is composed of a longitudinal bundle (ventral and dorsal) and a transverse bundle; during swallowing it contracts to shorten and medialize the pharynx, while during breathing it depresses the soft palate modulating the closure of the velopharyngeal, and acts as a sphincter for the nasopharynx area (separating the nasopharynx from the oropharynx) (Figure 2) [42,56,57]. PP is innervated by the lesser palatine nerve and the pharyngeal plexus [44]. In patients with OSA, the PP muscle fibers undergo a decline in their volume (atrophy), with increases in connective tissue (fibrosis); the muscle loses its contractile capacity [58]. As in other areas, there is a decrease in the oxidative capacity and in the number of capillaries of the contractile fibers [48].

**Figure 3:**
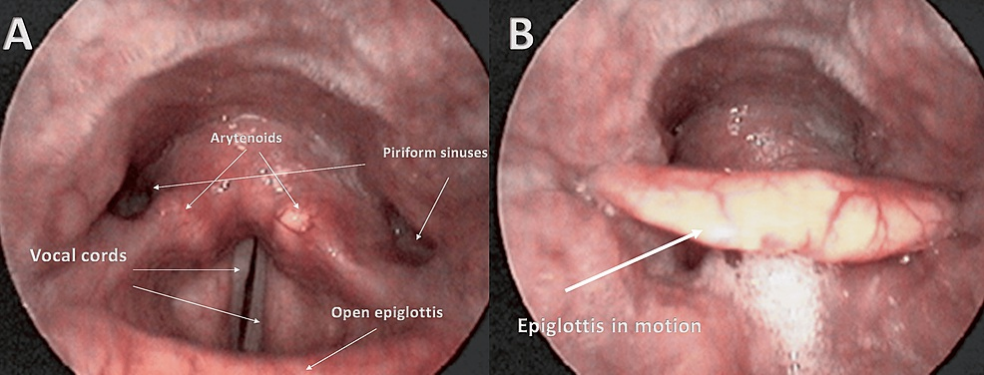
Photo A shows, through a fibrolaryngoscopy examination, the laryngeal area and some of its anatomical components; photo B shows the same area but with the epiglottitis toward the closure phase The photo is owned by Bordoni Bruno

The hypopharynx or laryngopharynx (below the oropharynx and behind the larynx) extends from the epiglottis to the lower limit of the cricoid; it is responsible for the passage of food to the passage of air and is a site of possible obstruction in patients with OSA [59]. This area could collapse due to various causes, such as the presence of macroglossia, decreased muscle tone, and hypertrophic lingual tonsils. The hypopharynx includes the posterior pharyngeal wall, pyriform sinuses, and the post-cricoid portion of the pharynx [59]. Below the hypopharynx, we can find the upper esophageal sphincter (UES) and the upper portion of the esophagus or the cervical esophagus [60]. The pharyngeal muscles include complex circular striated muscles of the outer layer: upper, middle, and lower pharyngeal constrictor. A fourth cricothyropharyngeus muscle arises from the cricoid arch until it crosses the inferior constrictor muscle, in particular, the cricolaryngeal portion; with loose connective bundles, it attaches to the lower horn of the thyroid gland and to the cricothyroid joint [61]. The external pharyngeal longitudinal muscles include the stylopharyngeal, salpingopharyngeal, and palatopharyngeal (previously described) [61]. The pharyngeal constricting muscles (PC), as regards the passage of air, are activated to increase the pharyngeal tone in the presence of negative pressure, avoiding the collapsibility of the pharyngeal tube: they manage the lumen of the pharynx [61]. The lower PCs are able to pull the hyoid bone posteriorly [39]. The transition area of the superior constrictor and the PP, at the level of the posterior-lateral area of the isthmus, is called Passavant's ridge; the latter is formed when the veil is closed during swallowing or phronesis [54]. The longitudinal muscles elevate and shorten the pharynx, fundamental actions in the swallowing process [62,63]. The actions of the longitudinal muscles can vary from subject to subject, according to their insertion, which can give a different vector force; in some people, these muscles can help the arytenoid muscle (vocal cords) during phronesis [64]. The pharyngeal and esophageal longitudinal muscles are in close anatomical communication, as is the coordination of the constricting and longitudinal pharyngeal muscles [55,65]. The superior constrictor merges with the buccinator and mylohyoid muscle through some fascicles and the lingual complex; the middle constrictor, in some subjects, shares an insertional portion on the hyoid bone with the mylohyoid muscle [42,66,67]. The pharyngeal muscles are innervated by the pharyngeal plexus via the vagus nerve and the glossopharyngeal nerve [39,44,62]. Neuromuscular incoordination is present in patients with OSA, and this involves the creation of a very complex clinical picture to visualize, without taking into account all the anatomical and functional relationships of the muscles mentioned. The space of the musculomembranous pharyngeal tube is reduced in OSA, compared to healthy subjects, for several reasons, including the posture of the occipitocervical area in extension and a more caudal position of the hyoid bone [16]. During sleep, the pharyngeal area is more prone to collapse, despite the possible electromyographic increase in the lingual complex; There is a lack of correct neurocoordination between the lingual complex and the peri-pharyngeal muscles, which are activated at lower electrical frequencies during the rest phase [68]. This is not a question of muscle fatigue but of a neuromotor dissociation between the tongue and peri-pharyngeal during sleep [69]. The collapse of the pharyngeal area may be more likely to occur during the last phase of exhalation [70]. The constricting muscles show structural protein and mitochondrial alterations and an increase in intramyocellular lipid droplets; there is an increase in apnea indices and an increase in such muscular dysfunctions [71]. In particular, in the upper constricting muscles, there is an increase in connective tissue (fibrosis), which increases contractile dysfunction; in the medium constricting muscles, there is a myopathy, typical of continuous remodeling from chronic injury, and an increase in anaerobic metabolism [72,73]. We do not know the specific adaptations of the lower constricting muscles. To the knowledge of the authors, we do not know in detail how the structure of the stylopharyngeal muscle and salpingopharyngeal muscle fits in patients with peripheral OSA. The laryngeal area can be a site of obstruction in patients with OSA due to a lack of coordination of the muscles that manage movement, and/or a decreased tone of the epiglottis (lax or ptotic), which can occlude the larynx. The thyroarytenoid, lateral cricoarytenoid, and interarytenoid muscles are able to close the glottis, through an adduction action; the posterior cricoarytenoid muscle (PCA) opens the glottis with an abduction action [74]. During the inhalation, the posterior cricoarytenoid raises the epiglottis (opens the glottis), reducing the resistance of the airflow; while, with the exhalation, the other three muscles contract to lower the epiglottis (closing of the glottis) [74]. With a deep inhalation, a fifth intrinsic laryngeal muscle contracts, the cricoarytenoid (CT) muscle; the latter stretches the vocal cords with minimal electrical activity in the phases of quiet breathing, but intervenes for deep inspiratory acts, allowing the anteroposterior laryngeal diameter to increase [74]. In this way, it increases the glottal area. CT is considered an accessory respiratory muscle and is activated in the presence of insufficient lung inflation and hypercapnia; CT is able to work in synergy with PCA to widen the glottal space to the maximum [75]. Cricothyropharyngeus muscle (CTP) during breathing helps the cricoid cartilage slide (forward and backward) and rotate, increasing the range of motion of the larynx and improving the movement of air flows [76]. The laryngeal and CTP muscles are innervated by the pharyngeal plexus [76]. Depending on the studies carried out on laryngeal obstruction, the percentages can range from about 12% to 23% of patients with OSA; the epiglottis would be longer in patients with glottal obstruction [77-80]. The collapse of the epiglottis can be classified as primary if the cause is the muscle itself, or secondary if the posterior tongue portion compresses the epiglottis and obstructs the larynx (Figure 3) [77].

Functional anatomy and adaptation of the lingual complex with OSA

**Figure 4:**
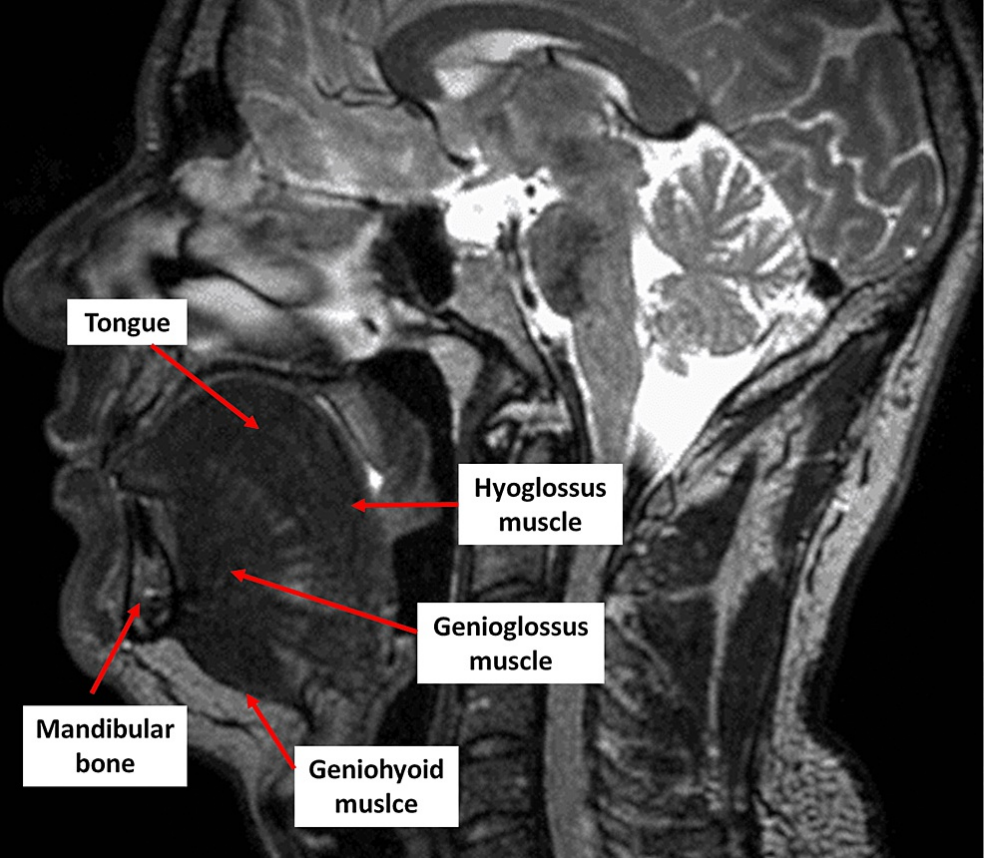
The image shows a magnetic resonance image of an obese subject; the size of the lingual complex is larger than in a non-obese subject The photo is owned by Bordoni Bruno.

The tongue is a finely coordinated muscular structure made up of intrinsic and extrinsic muscles. The intrinsic contractile districts (4 pairs) are transversalis, verticalis, inferior longitudinalis, and superior longitudinalis [81]. The extrinsic muscles (4 pairs) are genioglossus (GG), styloglossus (SG), hyoglossus (HG), and palatoglossus (PG) [81]. In the literature, it is possible to find two other muscles, such as the glossopharyngeus and chondroglossus, which are considered part of the extrinsic muscles [81]. The glossopharyngeus is a set of contractile fiber bundles deriving from the superior pharyngeal constrictor muscle; the chondroglossus derives from the hyoglossus muscle [81]. In the animal model, the superior and inferior longitudinal intrinsic muscles (and the genioglossus) are capable of producing a higher isometric force than the other lingual muscles; the intrinsic muscles are rich in glycolytic fibers and, in particular, in the anterior portion of the tongue, with a progressive decrease going towards the posterior [82,83]. As for size, the fibers of the intrinsic muscles possess a greater volume in the posterior portion, compared to the anterior of the tongue [83]. This organization could reveal different functions based on the organizational topography; finer and faster movements at the tip of the tongue and coarser and slower actions posteriorly [83]. In the aging process, it appears that the intrinsic muscles are spared from major phenotypic and morphological changes [83]. The superior longitudinal muscle (SL) in an animal model is active during inspiration while the posterior transverse muscle seems to play a role in maintaining the passage of airflow [83]. SL interdigitates with PG and with the outermost fibers of the HG; their connection allows for lateral lingual movement and retrusion [84]. The contraction of the SL allows the tongue to manage length during different actions [85]. The inferior longitudinal muscle (IL) has a close relationship with the lateral portion of the HG; both are coordinated by a dorsiflexion movement while, if IL is specifically activated, it is able to pull the lingual apex toward the posteriority and inferiority [84]. The fibers of the transversalis muscle (TV) control the thickness of the tongue and, in coordination with HG and PG, are activated by lateral retraction of the tongue [84,85]. The fibers of the verticalis muscle (V) are able to flatten the tongue laterally, to the right or to the left, and control the height of the tongue [84,85]. The tongue is considered a muscular hydrostat, that is, it not only keeps the volume constant, but the different movements carried out in the different functions are the result of the intervention of all the muscles that make up the tongue, in a percentage that is not synchronous [86]. It becomes almost impossible to distinguish specific activation in vivo of different lingual fibers and vectors [86]. Intrinsic muscles are active in maintaining the tonic for opening the upper airway in the presence of hypoxia (animal model) [87]. Currently, we do not know, specifically, the adaptations of intrinsic muscles in the presence of OSA. GG is the most studied muscle in the literature with OSA. GG is made up of type I and IIa fibers, which are interconnected on the horizontal (posterior area) and oblique (anterior area) planes, with an increase in volume going toward the posteriority; this arrangement allows the muscle to diversify the activation of the anatomical areas based on the action performed [82,88,89]. We must remember that the movement of the lingual complex is allowed due to all the muscles adjacent to the same tongue and not only to the GG [89]. GG is the most voluminous extrinsic muscle of the tongue; its fibers interconnect with all intrinsic muscles [89]. The actions of the GG are to protrude the tongue, depress the lingual body, perform retrusion of the apical portion of the tongue and ventroflexion, and stimulate the back of the tongue to form a convexity (useful for swallowing and phronesis) [89]. During the inhalation, GG pushes the hyoid bone forward to dilate the airways, with posterior-dorsal caudal traction and a subjective sensation of lingual retrusion [31]. GG activates approximately 250 milliseconds before it enters the airflow and before the movement of the diaphragm muscle, with a movement of about 2 millimeters in the inhale [90]. SG is the most lateral muscle of the lingual complex; with the posterior portion, it interdigitates with HG while with the anterior (wider), it connects with GG, HG, and IL [89]. When contracting, it allows the tongue to retrude and elevate the lingual lateral margin [89]. There does not seem to be a prevalence of a type of fiber (red or white), however, with advancing age, an increase in oxidative fibers may occur as compared to glycolytic ones [91]. HG appears to have a prevalence of glycolytic fibers, which decline with advancing age [91]. HG is activated in the phases of inspiration (animal model) [92]. In coordination with SG, it retracts the tongue, while activated alone and to the side, it depresses the lateral lingual portion [85,89]. The HG muscle is a thin district but interdigitates with all the muscles of the tongue [89]. PG shows a phenotypic balance of muscle fibers and a close relationship with the intrinsic muscle TV [53.89]. PG plays a role in the correct stimulation of lingual protrusion, improving the retropalatal opening [93]. Chondroglossus derives from the lesser cornu of the hyoid bone and is interdigitated with the intrinsic muscles of the tongue (in particular with IL) and merges with GG; working synergistically with and as HG [89,94]. The innervation of the tongue muscles is complex, and it is not always possible to have a clear distinction as to which nerve ending involves a specific contractile district. Each lingual muscle is made up of neuromuscular compartments (groups of fibers), which ensure different functions for the same muscle [95]. We must remember that the muscle complex of the tongue is made up of efferent and afferent fibers; the set of such neural information allows communication between the peripheral and central nervous systems. An important motor role is played by the hypoglossal cranial nerve (XII) while the sensory innervation is given by the glossopharyngeal nerve (IX), the lingual nerve (branch of the trigeminal nerve or V), the vagus nerve (X), part of the facial nerve (VII) and sympathetic branches from the superior cervical ganglion [39,95,96]. In patients with OSA, the lingual complex is enlarged and more voluminous, increasing resistance during inhalation; stiffness decreases or increases, depending on the study performed and with respect to non-OSA subjects [97]. These dimensional parameters do not seem to vary with the severity of the disease. The increase in volumes reflects the increase in adipose tissue within the muscles of the tongue, in particular, at the base [97]. The tongue suffers from apraxia and decreased stereognosis in patients with OSA, with an expressed strength that is less (measured by Iowa Oral Performance Instrument - IOPI) as compared to healthy subjects [98]. As mentioned, the GG is the most cited lingual district in research, as it plays an important role in the pharyngeal opening during inspiration, through lingual protrusion [99]. GG presents altered electromyographic responses, where it can be seen that in OSA, the muscle is less likely to mirror the presence of negative pressures (reflex for inspiration), both during sleep and during wakefulness [68,70]. There is a GG response latency, with a time of about 32 milliseconds, compared to healthy subjects with a latency time of about 24-26 milliseconds [70]. Despite the latency, the electrical spectrum of GG at rest and when awake is more active than in healthy subjects; the lack of ability of the muscle to dilate the upper airways correctly could depend on the incoordination between GG and the other contractile districts (electro-mechanical dissociation) [68]. The deformation of GG (movement) is less in patients than in subjects without OSA (less than about 1 millimeter on average); this reflects the presence of hypotonia (fibrosis, hypotrophy, phenotypic alteration, and myopathy), and the direct relationship between GG dysfunction and higher critical pressure (Pcrit) values [100,101]. In an animal model fed with many lipids, GG resulted in a numerical reduction of contractile fibers, a phenotypic imbalance (more white fibers), a reduction in mitochondrial function, and the presence of oxidation and apoptosis [102]. With OSA and advancing age, GG shows a lower myocellular repair capacity and more pronounced frailty in the face of hypoxia [103]. In obese patients and with OSA, GG is less prone to glucose uptake, with an altered metabolic environment (fewer resources for regeneration) and values of greater fatigue than in healthy subjects [104,105]. We have no specific data on other lingual intrinsic and extrinsic muscles in the presence of OSA. There is probably an electrical reduction in patients compared to non-OSA subjects [106]. Another muscle that attracts attention to obtaining a complete view of the muscles that allow correct breathing is the geniohyoid. The latter is not part of the lingual muscles; we find it below the GG, between the mandible and the hyoid bone [107]. The muscle is innervated by the hypoglossal nerve, probably via the anterolateral cervical atlanto-occipital plexus [108]. In patients with OSA, it may have an altered (dysfunctional) stiffness and a slowing down of electrical activity [99,106]. During the eupneic act (inhale), this muscle is active, albeit slightly, moving the hyoid bone forward, and is supposed to be useful for dilating the upper airways (Figure 4) [109].

Functional anatomy and adaptation of the diaphragm muscle with OSA

**Figure 5:**
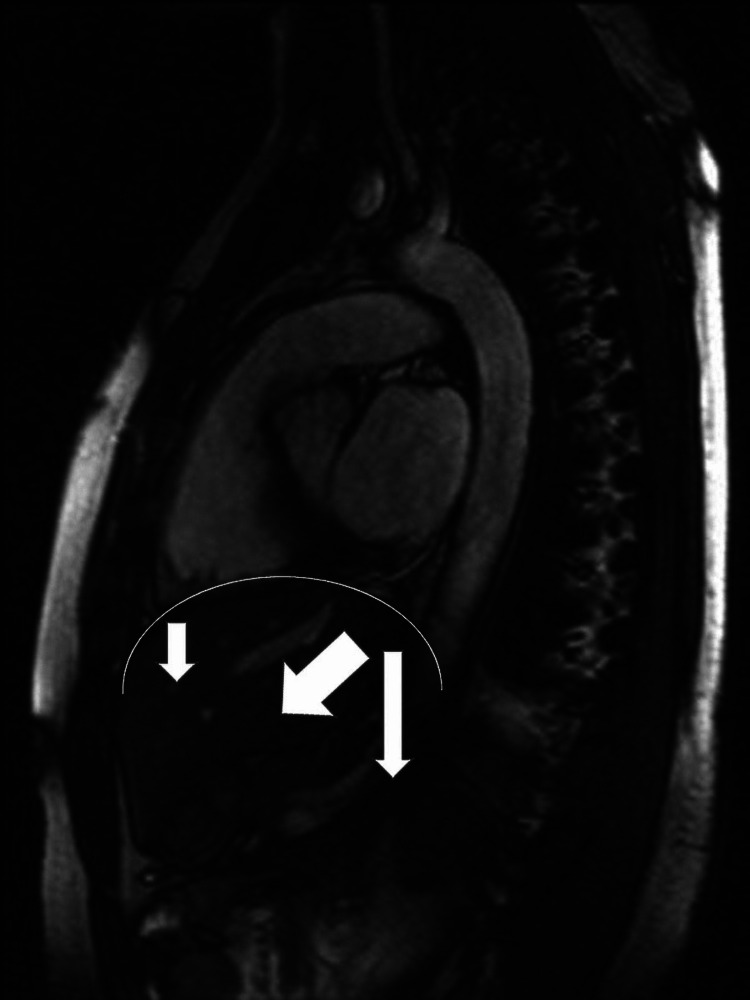
The sagittal magnetic resonance image shows the preferential directions of the diaphragm of a healthy subject before performing an inhalation, where the larger arrow highlights the oblique-anterior thrust, the longer posterior arrow highlights the greater excursion of the 'posterolateral area; the smaller arrow indicates the caudal movement of the anterolateral portion in a smaller percentage as compared to the posterior area The photo is owned by Bordoni Bruno.

The diaphragm muscle (DM) is the muscular boundary between the lower portion of the mediastinum and the upper portion of the abdomen. DM is the main muscle that allows eupneic acts while the other contractile districts are not directly able to guide the air inside the lung area but can facilitate or slow down its transit [39,110]. DM is asymmetrical, with a thickness and a vertical fiber position greater posteriorly, and thinner, with more horizontal contractile fibers anteriorly, with an average thickness of about 2-4 millimeters; this topographical disparity of the contractile fibers will affect the expression of movement [31]. DM remembers a mantle as morphology, with one side on the right raised by about 2 centimeters compared to the side on the left [31]. DM is crossed by multiple structures of the nervous, vascular, and muscular types: the sympathetic nervous system and vagus nerve; aorta and vena cava, superior epigastric vessels, thoracic duct, azygos and hemiazygos veins, lumbar venous plexus; and esophagus [31]. The portions of the DM involve the sternal area (xiphoid process), the vertebral area (from the thoracic vertebra 11 to the lumbar vertebra 4) through the pillars, and the costal area (the last 6 ribs) [31]. The main innervation comes from the phrenic nerve while the crural portion, where the esophageal hiatus resides, is innervated by the vagus nerve [31]. Before the DM, the dilator muscles of the upper respiratory tract are activated, thanks to the control of the CPG, allowing the entry of airflow. DM is rich in aerobic fibers, about 55% of which will be involved during a calm breath; the inspiration will determine the descent of the DM from a minimum of 2 centimeters to a maximum of approximately 10 centimeters [31]. The motion vectors during the inspiration will be caudal and oblique-anterior; specifically, the posterolateral area will move with greater emphasis (40% more than the anterolateral area), with an inclination of approximately 23.80° [31]. The anterolateral portion will have a movement with caudal and dorsoventrally direction; the right hemidiaphragm will have a smaller and slower range of motion than the left hemidiaphragm due to the presence of the liver (Figure 5) [31].

The vagus nerve manages the contraction of the esophageal hiatus so that during the actions of the rest of the DM's body, there is no antagonism for the passage of air and food [31]. One of the factors that determine the caliber of the airways is lung capacity and how they are filled (inflation) with the negative pressures created by the DM; if the DM is in dysfunction, the vital capacity decreases with an increase in the tendency of the dilator muscles not to intervene correctly, with a finding of the collapse of the upper airways. If intrapleural pressures are not present effectively (with a decrease in pulmonary inflation), the C-type lung afferent fibers send inhibitory information to the hypoglossal nerve; this mechanism will slow down the intervention of the lingual musculature, compared to the activation of the diaphragm, with the possible collapse of the airways [111,112]. If the relationship between DM and pulmonary inflation is not effective, there will be an increase in the finding of OSA [113]. Pressure intrathoracic is not directly related to AHI values, and airflow limitation is seen in many patients during daylight hours, to a greater extent than polysomnography evaluation alone [114]. Remember the importance of DM in patients with OSA could make a difference in the clinical setting. What do we know about the adaptation of the DM with OSA? DM appears to have a greater thickness in patients, through ultrasound measurements, but we do not know if this increase in size is related to hypertrophy (overcoming constant air resistance) or fibrosis (constant inflammation) [115,116]. An increase in thickness could be the result of greater constant contraction of the muscle due to constant obstructions of the upper airways. The literature is not always unique in indicating a decline in strength expressed by the DM; we do not know if there is actually fatigue of the central type (less neuromotor activation), or of the peripheral type (muscle metabolic alteration), or both. We know that there is a decline in the strength and endurance of DM in patients with OSA; probably, this condition could be linked to an inability to exhaustively recruit the motor units responsible for breathing [117-120]. We know that OSA is a systemic and not just a local disease, as demonstrated in the non-physiological adaptations of some peripheral muscles (anterior tibialis and quadriceps), where these muscles exhibit phenotypic, morphological, electrical, and metabolic alterations [117,121]. Stimulating DM with respiratory physiotherapy aimed at improving inspiratory musculature, decreases some parameters related to the diagnosis of OSA (AHI, Epworth sleepiness scale), making lung improvements recorded instrumentally (spirometry - forced vital capacity) [122,123]. DM is not only closely coordinated with the dilating muscles, during sleep and during wakefulness, but by stimulating the intervention (respiratory rehabilitation), it improves the symptomatological picture of the patient with OSA [58,123,124]. Another aspect that is hardly taken into consideration in the clinical setting related to DM and OSA is the vagus nerve, which innervates the diaphragmatic muscle area where the esophagus passes; the literature shows a reduction of the parasympathetic system in OSA [125]. This reduction is one of the causes that trigger cardiovascular disease and systemic inflammation [126]. We know that patients with OSA can suffer from nocturnal reflux and dysphagia, alterations attributable to a loss of synergy between the nerve phrenic and vagus nerve [127-129]. Stimulating the DM with breathing exercises improves the symptomatological picture related to gastric reflux and swallowing difficulties [130,131]. We do not know if exercises that stimulate DM are able to improve some symptomatic aspects, such as reflux and dysphagia, and the systemic response to inflammation. Evaluating how the DM behaves could be useful to indirectly understand the behavior of the CPG, and to highlight the neural dyssynergy of the respiratory muscles of the upper airways [132]. From the activity of the extra-diaphragmatic muscles (such as the alae nasi and GG), we cannot draw useful parameters to understand how the DM behaves; on the contrary, by examining the DM, we have useful clues to understand how the extra-diaphragmatic musculature is activated [132,133]. Considering that the DM is the main engine that allows the creation of negative pressures in the thoracic cavity and the entry of air into the lungs, allowing to obtain the mechanical and biochemical reflexes for the optimal management of the upper airway muscles, DM should have increased space of interest in the clinic with OSA patients. Many aspects of the pathology of OSA are not taken into consideration with respect to DM, both as an adaptation and as a rehabilitation. Further clinical and research efforts should be made to improve patient care management.

## Conclusions

Obstructive sleep apnea (OSA) is a sleep disorder leading to chronic intermittent hypoxia (CIH). Low values of chronic nocturnal desaturation negatively impact the psychophysical and social sphere of the patient, with an increase in health risks. These risks lead to cardiovascular pathologies, behavioral disturbances, and cognitive impairment, which require additional curative efforts. Most of the studies carried out to understand how the musculature of the airways behaves, looking for a therapeutic solution, largely focus on the genioglossus and other minor districts. There is hardly any clinical interest in the presence of OSA in the diaphragm muscle. This article reviewed the functional behavior and adaptation of the dilating musculature and the diaphragm muscle with evidence of OSA, highlighting the scarcity of clinical information on the latter muscle in the presence of chronic sleep apnea. We hope for a greater effort on the part of the scientific community in obtaining more information on the relationship between the diaphragm and OSA, with the ultimate aim of improving the curative approach.
